# The range and nature of reproductive health research in the occupied Palestinian territory: a scoping review

**DOI:** 10.1186/s12978-019-0699-4

**Published:** 2019-04-03

**Authors:** Aisha Shalash, Hasan M Alsalman, Alaa Hamed, Mai Abu Helo, Rula Ghandour, Loai Albarqouni, Niveen ME Abu Rmeileh

**Affiliations:** 1 Institute of Community and Public Health, Birzeit University, oPt, P.O.Box. 14, Birzeit, Palestine; 2 Obestrics and Gynecology specialist, Khalil Suliman hospital, oPt, Jenin, Palestine; 3 Obestrics and Gynecology resident, PMC hospital, oPt, Ramallah, Palestine; 4 Obestrics and Gynecology specialist, Al-Hiba IVF center and The Arab Care hospital, oPt, Ramallah, Palestine; 5 Center for Research for Evidence Based Practice (CREBP), Faculty of Health Science and Medicine, Bond University, Robina, Australia

**Keywords:** Scoping review, Reproductive health, Occupied Palestinian territory

## Abstract

**Background:**

In order to set research priorities for reproductive health in the occupied Palestinian territory, it is vital to know what current research has been done in the field of reproductive health. The purpose of this scoping review is to examine the range and nature of reproductive health research in the occupied Palestinian territory and to identify research gaps in the existing literature.

**Methods:**

We searched four databases: EMBASE, PubMed, CINAHL, and Popline. We included studies that: (i) are published (with an abstract); (ii) relevant to reproductive health; (iii) Palestinians living in Palestine; (iv) participants over the age of 15 years; and (v) restricted to human research. Three independent reviewers screened title and abstracts, and extracted data from included articles. We conducted quantitative and qualitative analyses.

**Results:**

Of 1025 titles and abstracts screened, 145 articles were included. 52 (36%) articles were conducted in community setting and 34 (24%) were conducted in hospitals. There were 5 (3%) experimental studies. 15 articles had more than one main theme; 160 subthemes overall were identified. The most frequently studied theme was labor and delivery (*n* = 19; 12%). One article discussed adolescent reproductive health and menopause while no articles discussed men’s reproductive health.

**Conclusions:**

91% of the research conducted is observational. The focus of reproductive health research was to understand the topic, community and providers’ perceptions and knowledge. Articles related to the quality of services were limited. It is also important to research the reproductive health of women outside of reproductive age, men, and adolescents.

**Electronic supplementary material:**

The online version of this article (10.1186/s12978-019-0699-4) contains supplementary material, which is available to authorized users.

## Plain English summary

To be able to better understand the different needs of the occupied Palestinian territory in regards to reproductive health research it is first important to take into consideration the research that has been published in hopes to reduce research waste. The purpose of this scoping review was to examine the range and nature of reproductive health research in the occupied Palestinian territory and to identify research gaps in the existing literature. The best way was to do a scoping review to systematically search databases for published articles. Our search found 1388 articles. Out of these articles 145 were found to be relevant in the inclusion criteria of 1) were journal articles; (2) had an abstract; (3) about reproductive health; (4) about Palestinians living in Palestine; (5) participants over the age of 15; and (6) human research. The majority of articles published were covering topics related to antenatal care, as well as labor and delivery. Although these were the most studied topics, they focused mainly on understanding the topic, community and providers’ perceptions and knowledge. Very limited articles addressed the quality of these services. Important topics that has found there are no published research articles were menopause, preconception, and psychosocial services. The purpose of the scoping review was to include all available research regardless of the quality. Further research can be done to assess the quality of the existing reproductive health research in the occupied Palestinian territories.

## Key points

1. Performing relevant research is important in translating research into practice.
2. Engaging health professionals in research helps with implementation of results.
3. More reproductive health articles are needed on marginalized groups.
4. These marginalized groups include adolescents, men, and menopausal women.

## Background

Research waste has been a top of discussion for decades. Goes as far back as the 1960s when the importance of reducing avoidable research waste was measured. Great strides have been made to help decrease the 85% medical research waste, but there is still a long ways to go [1].

There is global interest in helping countries conduct priority setting research [2, 3] and to bridge the gap between research results and policy formulation [4]. In a study that looked at the matching of researchers’ priorities and the interest of patients or health providers, it was found that out of 334 studies examined only 9 considered researcher priorities with the interests of patients or health providers [5]. In the occupied Palestinian territory (oPt), with the feedback of Palestinian reproductive health stakeholders including; doctors, policy makers and university researchers; it found priority research should focus on health systems involving reproductive health, pregnancy-related issues, and post-natal and maternal complications. Adolescent's sexual and reproductive health, men’s reproductive health, single women’s reproductive health were not reproductive health stakeholders’ main concern [6].

The occupied Palestinian territory has been under chronic occupation for more than 60 years. Such chronic crises conditions pose many challenges for building a sustainable health system on one hand and producing good quality research on the other hand [7]. As a result, research production has been mainly a response to emerging health issues. A recent review showed evidence of mismatching between the health burden of certain diseases/conditions and the number of published research reports on those diseases/conditions in the oPt. Cardiovascular disease, cancer, and maternal and neonatal deaths accounted for more than two-thirds of the total deaths in the oPt (67%), but were addressed only in (23%) in published articles [8]. A compilation of research produced in reproductive health in the oPt is not available, although there is an indication of an increase in the number of articles addressing public health and medical topics in the last years [9]. To understand the different needs of the occupied Palestinian territory in regards to reproductive health research it is important to take into consideration previously published research. By doing this, we better understand the research we have and what research is needed. The purpose of this scoping review is to describe the range and nature of reproductive health research in the occupied Palestinian territory and to identify research gaps in the existing literature.

## Methods

### Search strategy

We systematically searched (from conception till 6th June 2017) four databases, PubMed, EMBASE, CINAHL, and Popline, using search terms that were relevant to reproductive health (e.g., reproductive health, antenatal) and Palestinian regions (e.g., oPt). The PubMed search strategy is available as (Additional file 1). The other databases were searched using the same words and appropriate Boolean and truncation operators. Due to the interest in published scientific articles only, grey literature and unpublished reports were not included. Reference lists of all included studies were hand-searched to identify any additional relevant articles. Using Thomson Endnote software, duplicate articles were removed. There was No language restriction applied.

### Study selection

Two independent reviewers screened titles and abstracts of retrieved articles. Articles were considered for inclusion if they meet our eligibility criteria: (1) were journal articles; (2) had an abstract; (3) about reproductive health; (4) about Palestinians living in Palestine; (5) participants over the age of 15; and (6) human research. An age limit of 15 and over was used because reproductive age starts at 15 for both males and females. It is important to note that although articles only conducted in Palestine were included, some articles were multi-country studies and these studies were considered eligible for inclusion. Discrepancies were discussed among the reviewers and when consensus could not be reached a third reviewer made the final decision of inclusion or exclusion.

### Data extraction

Two independent reviewers extracted data on: title, year of publication, journal name, impact factor of journal, number of citations, number of authors, co-authors, first author affiliation, at least one author has Palestinian affiliation, and funding sources if mentioned. Further, we extracted data on: geographical location, setting of study, study population, age of population, sample size, study design, purpose of study, and theme of study, outcomes, and key findings. Discrepancies were discussed among reviewers and a third reviewer made the decision if discrepancies could not be resolved. Full text articles were excluded if after thoroughly reading the article did not fall under the inclusion criteria of the title and abstract screening.

### Data analysis

The results of article characteristics were summarized using frequency and percentages. Each article was also analyzed thematically looking for themes and subthemes by two independent researchers. An article was not limited to one theme or subtheme. The themes and subthemes were discussed by two researchers and three local gynecologists agreed on the results presented in this paper.

## Results

**Fig. 1:**
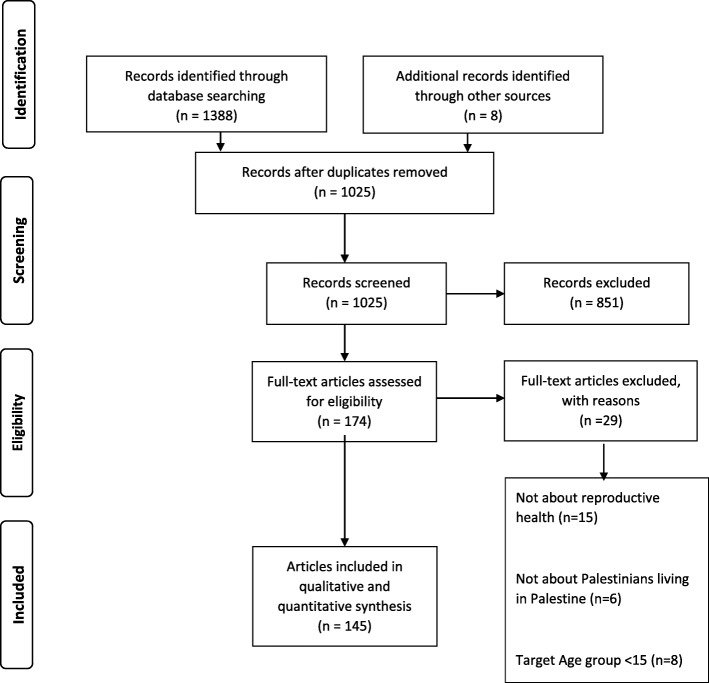
Flow diagram of search and study inclusion and exclusion process

The initial database searches yielded a total of 1388 articles. Of 1025 articles screened, 174 articles were eligible for full text screening. Of these 174 articles, 145 articles were included for qualitative and quantitative analysis (Fig. 1). The 29 articles were excluded because during full text screening they were found (*n* = 15) not to be about reproductive health, (*n* = 6) Palestinians living in Palestine and (*n* = 8) the target age group was less than 15. Article characteristics are available in (see Additional file 2).

### Study characteristics

Included articles were published between 1986 and 2017 (72 articles were published between 1986 and 2009, and 73 articles 2010–2017). 86 articles were published in general health or medical journals (59%) compared to 59 published in journals specific to reproductive health and gynecological medicine (41%). The included articles were published in 111 different journals, with the more popular journals being Eastern Mediterranean Health Journal (*n* = 9), BMC Pregnancy and Childbirth (*n* = 4) and Reproductive Health Matters (n = 4) and Health Policy (*n* = 3), Journal of Interpersonal Violence (n = 3), and Social Science & Medicine (n = 3). 14 journals have two publications and the rest with only one each. Impact factor of these journals ranged from no impact factor to 44; with the median impact factor being 2. Article citations spanned from 0 to 233 citations, with 20% of the articles having no citations and 80% of the articles with 24 citations or less.

Palestinian first authors wrote articles (69/145; 48%) of the time, while (75/145; 52%) of first authors were from Israel, the Region or International authors. First authors that were from Israel, the Region or International affiliation, (50/75; 67%) did not include at least one Palestinian co-author on their published article. 70 (48%) articles reported funding sources; 19 (13%) were from Europe, 16 (11%) were from North America, and 18 (12%) articles were locally funded or received no funds. Table 1 describes the general characteristics of included articles in this study.

**Table 1:** Characteristics of included articles

	N(145)	%
Publication Year		
1986–1989	2	1.4
1990–1999	7	4.8
2000–2009	63	43.5
2010–2017	73	50.3
Journal Type		
General	86	59.3
Specialized	59	40.7
Journal Impact Factor		
Less than 1	19	13.1
1 to 3	82	56.6
Greater than 3	15	10.3
No Impact Factor	29	20.0
Affiliation of First Author		
Local	69	47.6
Israel	17	11.7
Regional	20	13.8
Europe	23	15.9
North America	10	6.9
Asia	5	3.4
Unknown Affiliation	1	0.7
One of the Co-authors has Local Affiliation		
Yes	94	64.8
No	50	34.5
Unknown Affiliation	1	0.7
Funding of Journal Article		
Local	8	5.6
Israel	1	0.7
Regional	9	6.2
Europe	19	13.1
North America	16	11.0
UN	7	4.8
No funding received	10	6.9
Not mentioned	75	51.7

Going into more depth of the journal article it showed that most studies were conducted in the West Bank alone (58/145; 40%). Journal articles that included oPt in a multi-country study about reproductive health were (6/145; 4%). The majority of the journal article were conducted in the community setting (57/145; 39%), only (37/145; 25%) were conducted in hospitals and (41/145; 28%) were conducted in a private or government clinic. There were 5 (3%) experimental studies conducted in reproductive health in the oPt. Over half of the studies (84/145; 58%) were cross-sectional studies and (16/145; 11%) were case control studies. More details of the breakdown can be found in Table 2.

**Table 2:** Study characteristics of included articles

Region of Study Population	N (145)	Percent
Occupied Palestinian territory	36	24.8
West Bank	58	40.0
Gaza Strip	36	24.8
Regional	9	6.2
International	6	4.1
Setting of Journal Article		
Hospital	33	22.7
Hospital and Clinic	4	2.8
Clinic	41	28.3
Community	57	39.3
Prison	1	0.7
Not applicable (N/A)	9	6.2
Study Design of Journal Article		
Interventional/Experimental Studies	5	3.4
Observational Studies		
Cross-Sectional Study	84	57.9
Case Control	16	11.1
Cohort Study	14	9.7
Qualitative Study	15	10.3
Case Series/Case Report	3	2.1
Literature Review	8	5.5

### Main themes and subthemes

Some of the articles (*n* = 15) had more than 1 main theme and so 160 subthemes overall were identified. The most published theme was labor and delivery (*n* = 19). The subthemes involved were breastfeeding, cesarean section, childbirth, stillbirths, amniotomy, and vaginal examination during birth. Breastfeeding was explored in the sense of factors that affect breastfeeding and cesarean section mainly looked at percentages within hospitals and communities. Childbirth was one of the main subthemes concerning labor and delivery. Many of the articles discussed routine services, safe place and location for delivery, practices, evidence into practices taken, quality of care, and labor management practices. Stillbirths mainly estimating numbers and vaginal examination during birth looked at women’s feelings and knowledge about examination.

The second most published themes were both contraception and postnatal care (*n* = 16). Within contraception, fertility was the most studied. Articles looked at the changing trends in fertility, the value of children, economic, political, social and etiological factors that were associated with fertility. The knowledge, services, practices, and gender relations of family planning were also present as subthemes under contraception. Postnatal care also had a wide variety of subtopics. Topics such as breastfeeding, postnatal services, pregnancy intentions, Trichomonas Vaginallis and one article on postpartum depression. With breastfeeding, articles discussed the use of breastfeeding as a contraception method and its effectiveness, the marketing of breastmilk substitutes, lead levels in breastmilk, and the prevalence and effect of breastfeeding prevalence on infant’s health outcomes. Postnatal services discussed were the factors associated with the lack of postnatal care. Postpartum depression was discussed briefly in an article that looked at the quality of life postpartum.

Antenatal care can be found to be the theme in 14 of the journal articles. The articles found had a variety of subthemes. Three articles discussed pregnancy intentions, birth spacing, and preterm birth and its risk factors. Three articles explored the notion of maternal serum screening and the prevalence of cytomegalovirus and toxoplasmosis. Two articles sought to look at the consumption of oral drugs while pregnant. One article can be found on the prevalence of anemia among pregnant women. There were then a number of articles that were case studies or case-control studies that discussed Walker-Warburg syndrome, a successful pregnancy in a patient with Takayasu’s arteritis, and exposure to indoor pollution. No articles were found to discuss preeclampsia, antenatal coverage or services, treatment of STIs, screening of mental health and other issues, and nutritional behaviors while pregnant.

Gender-based violence had (*n* = 13) a variety of subthemes that could be found in the articles included in this review. Three articles discussed political violence and its effect on intimate partner violence. Many articles looked at the knowledge and perceptions of wife abuse in society and the prevalence and perceptions of domestic violence in Palestinian society. One article discussed the effects of virginity testing and another on the domestic violence against single-never married women. No articles discussed gender-based violence services that are available to women.

Reproductive system cancers were themes in 11 different articles. The main subthemes discussed were the knowledge and perceptions of women of breast cancer, barriers to early detection of breast cancer and women’s experience coping with breast cancer. There were articles that discussed the genetic mutations of the breast cancer gene and only one article discussed ovarian cancer. No articles discussed reproductive health cancer services or cervical cancer.

Sexually transmitted diseases (STDs) could be found 10 times as main themes. Six articles discussed HIV/AIDs. Out of these six articles, four involved the prevalence of HIV/AIDs in drug users and prisoners. The other two articles discussed the knowledge and attitudes among women towards HIV/AIDs. The other articles discussed the prevalence and trends of Trichomonas Vaginallis, Chlamydia Tractomatis and Hepatitis C Virus (HCV) risk factors and genotypes. No article discussed STDs and the available services. Some of the main themes that were not covered in published articles were adolescent health, menopause, psychosocial services, preconception, and men’s reproductive health. An extended list of main themes and subthemes can be found in Table 3.

**Table 3:** Main Themes and Subthemes Presented in Journal Articles (*N* = 160)

Main Themes	Number of articles	Sub Themes
Labor/Delivery	19	Breastfeeding, Cesarean Section,Childbearing, Childbirth Stillbirths, Amniotomy, Vaginal Examination during Birth
Contraception	16	Family Planning, Fecundability, Fertility
Postnatal Care	16	Breastfeeding, Pregnancy Intentions, T. Vaginallis, Postnatal Services, Postpartum Depression
Antenatal Care	14	Antenatal Services, Pregnancy Intentions, Cytomegalovirus, Early Pregnancy, Herb Use, Pregnancy, Pregnancy Risk Factors, Preterm Birth, Toxoplasmosis, Exposure to Secondhand Smoke
Gender-based Violence	13	Domestic Violence, Political Violence, Sexual Abuse
Reproductive System Cancers	11	Breast Cancer, Ovarian Cancer, Cancer Mortality
STDs	10	Chlamydia, HCV, HIV, Infertility, T. Vaginallis, AIDS, HBV
Infertility	9	Adolescence, Genetics, IVF, Genetics, Ovarian Hyperstimulation, PCOS
Maternal Morbidity	9	Anemia, Gestational Diabetes, Hypertension, PCOS, Preeclampsia, Maternal Deaths, Diabetes
Health Information, Communication and Education	6	AIDS, Health Awareness, Health Education, Maternal Education, Maternal Health
Marriage	6	Consanguinity, Early Marriage
Congenital Disorders	4	Beta-Thalassemia, Congenital disease
Quality of Services	4	Antenatal Services, Childbirth, Maternal Near Miss, Mortality Data, Postnatal Services
RTIs	4	Chlamydia, Pregnancy infections, T. Vaginallis, HPV
Miscarriages	3	Miscarriages, Recurrent Pregnancy Loss
Nutrition	3	Herb Use, Iron Supplements
Preconception	3	Family Planning, Genetic Counseling, Genetic Testing
Gender and Rights	2	Premarital Law, Never Married Women
Psychosocial Services	2	Maternal Health, Mental Health
War and Conflict	2	
Adolescents Reproductive Health	1	Sexual Risk
Menopause	1	Midlife
Never Married Women	1	Mortality Patterns
Service Delivery and Coverage	1	Quality of Antenatal Care

## Discussion

### An increase in reproductive health research production

This scoping review summarizes the range and nature of the available published reproductive health articles in the occupied Palestinian territories and identifies the types of research available as well as research gaps. Every year after the year 1999, there was at least one reproductive health article published from the country. Half of the articles published about reproductive health in the oPt came 10 years after the International Conference of Population and Development (ICPD). Realizing that in order to further the progress of the Millennium Development Goals (MDGs) and now the Sustainable Development Goals (SDGs) related to reproductive health, there is still a need for more investment in reproductive health policies and education. All countries in the year of 2005 recommitted to making further progress in the ICPD Program of Action [10]. The world’s recommitment to reproductive health showed the topic to be of interest and can explain the rapid increase in publications after 2005.

### Type and quality of studies

Further, the types of studies conducted were limited to observational studies. This is not unique to reproductive health research as recent review of public health research revealed that more than 75% of published research in the oPt is observational research [9]. There is almost a complete absence of operational and/or implementation research. The introduction of implementation research in the developing countries has been encouraged recently but still funding allocated to such research is limited [11].

### Most studied topics

The majority of articles published were covering topics related to antenatal care, as well as labor and delivery. Although these were the most studied topics, they focused mainly on understanding the topic, community and providers’ perceptions and knowledge. Very limited articles addressed the quality of these services; although it was ranked as top research priority area by the Palestinian health providers working in reproductive health [6]. Such research is needed especially for fragile health systems that function under humanitarian settings where women and children are the most affected groups [12]. The sustainability and scaling up of effective interventions and programs are core components that need to be further researched in crises conditions [13].

### Least studied topics

Adolescent sexual and reproductive health and rights (ASRHR) was only discussed in one article. The lack of published articles on this topic is not surprising. The priority setting meeting of RH professionals in the oPt showed that ASRHR was not considered to be a topic of high demand [6]. Limited research on ASRHR was found in the Arab countries as well [14] and some Arab countries still do not consider ASRHR a priority [15]. Worldwide there have been a number of activities aimed at promoting ASRHR but have faced challenges in sustainability. The Global strategy for women’s, children and adolescents’ health (2016–2030), has encouraged research in this area [16, 17].

Adolescent sexual and reproductive health and rights is a topic in development without the cultural and religious beliefs that the oPt and other Arab countries face [18]. Many adolescents believe the Maternal and Child Health clinic are for married men and women only. Cultural and religious beliefs play a huge role in the lack of education and information provided to unmarried Arab men and women [19]. Sex before marriage and abortion are also two topics that are of cultural and religious sensitivity. Both topics are of importance; but due to cultural and context specific values, we have no numbers of actually how often they occur in Palestinian society [18]. It is estimated that more than one million unsafe abortions occur in the Middle Eastern and North African region a year [20]. We need special study designs that take into consideration cultural beliefs. Such capacity is lacking in the Middle East and North Africa in general and in the oPt as well [19].

No published research articles were found on important topics such as; menopause, preconception, and psychosocial services. The global strategy adopted the life course approach targeting health and well-being at every age [17]. It will be vital to encourage the research of women after they are no longer of reproductive age especially for their menopause transition [21], non-communicable diseases, well-being and quality of life [22]. Reproductive cancer was also a marginalized topic with only screening, knowledge, practices and screening barriers were addressed mainly for breast cancer. Little information was available for ovarian or cervical cancer.

### Health system priority and need

The active engagement of health providers in research was not observed and the translation of research results into practice were not documented in the reviewed articles. Although the oPt had more than 20 years screening program for cervical cancer using the pap-smear, there were no publications evaluating its effectiveness or benefit. There are several informal reports that were produced evaluating program specific reproductive health services (the pap smear screening program) but are not available for the public.. If health professionals are involved in the process of priority setting, research becomes more valuable and research waste is reduceable [23].

### Strengths and limitations

This scoping review was the first to bring together scientifically published research on reproductive health in the occupied Palestinian territory. Using this review, we can better prioritize in which reproductive health research is essential. We can also use this information to better understand the reproductive health research status of the oPt.

In the current study, we tried to give readers a broad scope of the nature and range of topics published on Palestinian reproductive health. For this purpose, one of the limitations that can be found was that grey literature was not searched given we were interested in published scientific articles. There are several informal reports that were produced evaluating program specific reproductive health services but are not available for the public, for example the pap-smear screening program because no results were published.. The current review focuses on the content of the article rather than the quality of the research published. The purpose of the scoping review was to include all available research regardless of the quality. Further research can be done to assess the quality of the existing reproductive health research in the occupied Palestinian territories.

## Conclusion

Reproductive health research in the past 20 years has increased in the oPt. The oPt has made great strides in trying to perform relevant reproductive health research. It is unclear if this research has actually translated into practice. The results of this paper can be used to advocate for reproductive health research that is not of duplicate nature and is a priority to reproductive health professionals. It is vital to take into consideration the needs of the community and engage reproductive health professionals in the research process from setting the research priorities into translating research results into change in policies or practices. The formation of multi-disciplinary teams will allow for different people of different backgrounds and expertise to give diverse ideas to make sure the research is comprehensive in satisfying all needs for the people involved.

## Additional files

Additional file 1: Search terms and strategy. (DOCX 14 kb)

Additional file 2: Article Characteristics of included articles. (XLSX 27 kb)
