# Supplementary material for: The range and nature of reproductive health research in the occupied Palestinian territory: a scoping review
Source: Reprod Health. 2019 Apr 3;16:41. doi: 10.1186/s12978-019-0699-4 (PMC6448219; doi:10.1186/s12978-019-0699-4)
Supplement: Supplementary file 1 — Search terms and strategy. (DOCX 14 kb) [file 12978_2019_699_MOESM1_ESM.docx]

| **Additional File 1: Search Terms and Strategy** | |
| --- | --- |
| **Population Terms:**   1. Gaza 2. Gaza Strip 3. East Jerusalem 4. Occupied Palestinian Territory 5. West Bank 6. Palestine 7. Palestinian Authority | |
| **Reproductive Health Terms:**   1. Reproductive Health 2. Pregnancy 3. Abortion 4. Miscarriage 5. Delivery 6. Postpartum 7. Family planning 8. Contraception 9. Infertility 10. Women’s Health 11. Maternal Health 12. Prenatal 13. Postnatal 14. Antenatal 15. Breast cancer 16. Ovarian cancer 17. Uterine cancer 18. Cervical cancer 19. AIDs 20. HIV 21. STI 22. Sexually transmitted diseases 23. Reproductive tract infections 24. Sexuality 25. Menopause | 1. Anemia 2. Nutrition 3. Breast feeding 4. Lactation 5. Labor 6. Sterilization 7. Fertility 8. Subfertility 9. Preeclampsia 10. Eclampsia 11. Menarche 12. Menstrual cycle disorder 13. Pelvic inflammatory disease 14. Gynecological tumors 15. Childbirth 16. Genitourinary tract infections 17. Maternal mortality 18. Cervicitis 19. Climacteric 20. Domestic violence 21. Intimate partner violence 22. Gender based violence 23. Obstetric |
| **Search Strategy Used:**  (Gaza or "Gaza Strip" or "East Jerusalem" or "Occupied Palestinian Territor*" or "West Bank" or Palestine or "Palestinian Authority" ) AND  ("Reproductive health" or pregnanc* or abortion* or miscarriage* or delivery or postpartum or "family planning" or contracept* or infertility or "women's Health" or "maternal Health" or prenatal or postnatal or antenatal or "breast cancer" or "ovarian cancer" or "uterine cancer" or "cervical cancer" or AIDs or HIV or STI or "sexually transmitted disease*" or "reproductive tract infection*" or sexuality or menopause or anemia or nutrition or "breast feeding" or lactation or labor or sterilization or fertility or "sub fertility" or preeclampsia or eclampsia or menarche or "menstrual cycle disorder*" or "pelvic inflammatory disease*" or "gynecological tumor*" or childbirth or "genitourinary tract infection*" or "maternal mortality" or cervicitis or climacteric or "domestic violence" or "gender based violence" or "intimate partner violence" or obstetric*) | |
